# The Predictive Value of Tagalog Voice Morphology in Filler-Gap Dependency Formation

**DOI:** 10.3389/fpsyg.2020.00517

**Published:** 2020-04-15

**Authors:** Jed Sam Pizarro-Guevara, Matthew Wagers

**Affiliations:** Department of Linguistics, University of California, Santa Cruz, Santa Cruz, CA, United States

**Keywords:** active dependency formation, filler gap dependency, voice morphology, cue validity, Tagalog

## Abstract

Research has shown that when processing filler-gap dependencies, comprehenders do not wait until they encounter all of the bottom-up information in the input. Instead, they use various types of linguistic information to predictively posit a gap that would allow the dependency to be resolved. They can use syntactic (Traxler and Pickering, 1996), lexical (Trueswell et al., 1994), morphological (Kamide et al., 2003), and prosodic (Nagel et al., 1994) information. Here we examine whether Tagalog comprehenders use the language's voice morphology to guide their incremental interpretations. We hypothesized that voice allows comprehenders to commit to an interpretation upon encountering the verb, since they have information about the event structure at this point in time and by virtue of the voice morphology, the thematic role of the filler. In experiment 1, using an acceptability judgment study, we found that comprehenders differed in how they used the different voices in different filler-gap contexts to detect the licitness of displacements. These differences may have consequences for how voice is used in real-time. In experiments 2 and 3, using the stops-making-sense paradigm (Boland et al., 1990), we found that comprehenders used voice as a cue to actively associate the filler with the gap. However, in experiment 3, the way in which they used voice varied by type and varied across types of filler-gap dependencies. We argue that comprehenders were using construction-specific cue validities when processing filler-gap dependencies. However, they also engaged with other classes of linguistic information, including (but not limited to) information about the structural similarities and the thematic complexity of the dependencies involved, and the relative frequency of the different types of voices in the language. These interactions resulted in processing asymmetries.

## 1. Introduction

Comprehending non-local dependencies in real-time is challenging to comprehenders because of incremental uncertainty. For example, the sentence in (1) is temporally ambiguous. At the verb *read*, the noun *book* can function as the direct object of *read*, as in (1-a), or as the object of the preposition *of* , as in (1-b). From the point of view of comprehenders, both sentences are string-identical until they have encountered the elements after *read*. Only after encountering *was* do they have indefeasible evidence that *book* is the direct object of *read* in (1-a). Only after *a review of* do they know that it cannot be the direct object and only after *was* do they know that it is the prepositional object in (1-b).


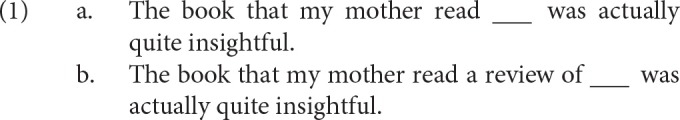


How do comprehenders behave when faced with incremental uncertainty? One possibility is that they wait until all of the bottom-up information becomes available. Another possibility is that they actively construct dependencies even in the absence of the bottom-up information needed for disambiguation. In this paper, we look at filler-gap dependencies, a specific type of non-local dependency. We add to the body of literature supporting active dependency formation, as opposed to waiting for evidence before constructing the dependency.

Filler-gap dependencies are constructions where a constituent appears not in its thematic position, but rather to the left of the verb it is associated with. Consider (2-a): the noun phrase *the defendant's assets* is in its thematic position, after the verb *confiscated* where direct objects generally appear. Now contrast this with (2-b)–(2-c): no noun phrase appears after *confiscated* even though one would normally expect to find one in this position. Instead, the corresponding noun phrases have been displaced, and now occur before the verb. Throughout, we refer to the displaced noun phrase as the filler.


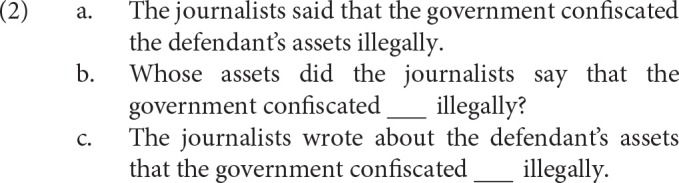


Even though the fillers have been displaced, comprehenders still interpret them as the direct object of the verb. That is, comprehenders seem to understand the fillers to correspond to an empty position after the verb. Throughout, we refer to this empty position as the gap, and represent it as “” above. The relationship between the filler and the gap is what we refer to as a filler-gap dependency.

When comprehending filler-gap dependencies in real-time, comprehenders are also faced with incremental uncertainty about how to associate the filler and the gap prior to encountering all of the bottom-up evidence from which to infer the position of the gap. What classes of linguistic information do comprehenders use as cues to guide their interpretations of these dependencies? Studies have shown that they can leverage different classes of information, ranging from syntactic (Stowe, 1986) to prosodic (Nagel et al., 1994). In this paper, we investigate the role of Tagalog voice morphology in comprehending filler-gap dependencies. Tagalog is a verb-initial Austronesian language spoken primarily in the Philippines by ~22.5 million native speakers (Philippine Statistics Authority, 2017). Voice morphology always cross-references the argument marked with *ang* [aŋ] in the clause and it is intimately correlated with which argument can participate in filler-gap dependencies. Thus, voice could provide Tagalog comprehenders an early and reliable source of information about how to associate the filler and the gap.

The present study has two major goals. Our first goal is to better understand the role of Tagalog voice morphology in the incremental processing of filler-gap dependencies. In order to do so, we asked the following questions: (i) is voice morphology a cue that Tagalog comprehenders use in real-time? (ii) if it is, are the different types of voice used the same way? and (iii) is voice used the same way across different types of dependencies? We believe that it is always an empirical question what classes of information comprehenders actually use as cues and how these cues are used in real-time sentence processing. We know from the grammatical illusion literature—like agreement attraction (Clifton et al., 1999; Pearlmutter et al., 1999; Wagers et al., 2009) and illusory licensing of negative polarity items (Drenhaus et al., 2005; Xiang et al., 2006; Vasishth et al., 2008), to name a few—that comprehenders are sometimes sensitive to irrelevant cues. By irrelevant, we mean that these are cues that are not instantiated by the dependency of interest. Furthermore, comprehenders can also ignore very reliable cues and at times, even do so in favor of less reliable ones (MacWhinney et al., 1984; Gagliardi and Lidz, 2014). Thus, even if voice is a cue that is available in the input and is generally reliable in determining the identity of the filler, whether Tagalog comprehenders actually use it remains an open question. How they will use it is another open question.

Our second goal is to contribute to the growing efforts to incorporate data from “smaller” languages in psycholinguistic research. This enterprise has been referred to as field psycholinguistics (Christianson and Cho, 2009) or cross-linguistic psycholinguistics (Norcliffe et al., 2015). Much of what we know about sentence processing suffers from a lack of linguistic diversity, since the data come primarily from Western (and some East Asian), educated, industrialized, rich, and democratic language users (“WEIRD”; Henrich et al., 2010). In fact, only 10 languages make up the bulk (~85%) of the research on language processing (Anand et al., 2011). Despite the fact that the Austronesian language family accounts for 5–17% of the world's linguistic diversity (Eberhard et al., 2019), it is “virtually absent from psycholinguistic research” (Wagers et al., 2015). Thus, the present study is of theoretical import because it addresses directly this lacuna by investigating a heavily under-represented language (family)—one that is typologically different from the more familiar languages that are well-characterized in the psycholinguistics literature.

In recent years, there has been a slow but steady accumulation of psycholinguistic work focusing on typologically diverse languages. For a review, see Norcliffe et al. (2015). Relevant to the second goal of the present study are five studies on Tagalog sentence comprehension. Sauppe (2016) used the visual word paradigm to investigate whether Tagalog comprehenders anticipated the referents of upcoming post-verbal arguments based on syntactic function or semantic role in canonical declaratives (i.e., verb-initial sentences). Meanwhile, Garcia et al. (2019b) used self-paced listening with picture-selection and eye-tracking while listening (Garcia et al., 2019a) to investigate whether comprehenders leveraged word order and morphosyntactic markers (i.e., case and voice) in the comprehension of canonical declaratives. The present study differs from the previous studies in two ways. First, the previous studies have visual cues available to the comprehender that could give them an overview of the global event structure; ours do not. Second, our experimental items involve filler-gap dependencies; theirs did not. The relative simplicity of their experimental items, coupled with a rich paralinguistic context, might reduce the need for comprehenders to deploy resources from other parts of the grammar. What the present study is interested in is whether voice will be deployed as a cue in contexts where an argument is displaced from its thematic position and has to be held in working memory. Bondoc et al. (2018) and Tanaka et al. (2019) used a referent-selection task, a modified version of a picture-selection task, to investigate the comprehension of relative clauses in neurotypical and aphasic populations, respectively. While these studies do involve contexts where an argument is displaced from its thematic position, their results are based on interpretations that are the end state of comprehension. What the present study is interested in is not the final state of comprehension but rather the incremental choices comprehenders make as more of the input becomes available.

In the present study, we established that voice morphology is a cue that Tagalog comprehenders attend to and use when processing filler-gap dependencies in real-time. We added to the existing literature supporting the view that comprehenders actively construct dependencies even in the absence of disambiguating information. Crucially, we did so using a language that is heavily under-represented in the psycholinguistics literature. We also provided evidence that the way in which comprehenders used voice as a cue may be modulated by different classes of linguistic information, including (but not limited to) finer-grained information like construction-specific cue validities, and information about the structural similarities or the thematic complexity of the constructions involved and the relative frequency of the different types of voice. The interactions of these classes of information lead to processing asymmetries.

## 2. Background

In this section, we first provide an overview of the types of evidence used in the literature to support the idea that comprehenders do not wait until all of the bottom-up information becomes available, but instead actively construct dependencies by using various cues in the input. We then provide a description of Tagalog and the language's morphosyntactic properties that figure in our experiments. Finally, we present a hypothesis of how Tagalog comprehenders could use voice to actively construct filler-gap dependencies.

### 2.1. Comprehenders Actively Construct Filler-Gap Dependencies

Filler-gap dependencies challenge comprehenders to associate the filler with a gap, whose position is not always unambiguously indicated by the evidence in the input. Nevertheless, comprehenders do not wait until all of the bottom-up information becomes available (Crain and Fodor, 1985; Frazier, 1987; Frazier and Clifton, 1987). Instead, they attempt to establish this link by predictively positing a gap at each available position that would allow the dependency to be resolved without violating more abstract grammatical constraints like islandhood (Traxler and Pickering, 1996; Wagers and Phillips, 2009).

One piece of evidence supporting active dependency formation is the filled-gap effect (Crain and Fodor, 1985; Stowe, 1986). For example, Stowe (1986) found that reading times at the direct object pronoun *us* were longer in embedded *wh*-questions, like (3-a), than in minimally different embedded polar questions, like (3-b). This suggests that comprehenders posited a gap in direct object position after encountering the verb *bring* in (3-a), without waiting to check whether or not it was already occupied. Upon encountering *us*, their initial predictions were disconfirmed, as indicated by the longer reading times at this region in (3-a).


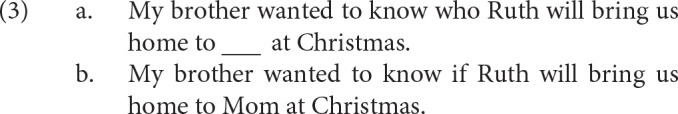


Another piece of evidence for active dependency formation is the plausibility mismatch effect (Garnsey et al., 1989; Boland et al., 1995; Traxler and Pickering, 1996). For example, Boland et al. (1995) found that participants judged the filler *which prize* in (4) to be implausible upon encountering the verb *visit* using the SMS paradigm, a self-paced word-by-word reading combined with an explicit acceptability judgment task. The filler *which prize* is implausible only if it is associated with the verb *visit*, as in (4-a). The pound sign (“#”) indicates implausibility. However, it can be plausible if it is associated not with *visit* but with a different verb downstream, as in (4-b). The comprehenders' judgment suggests that they were attempting to resolve the dependency prior to the identification of the gap's location.


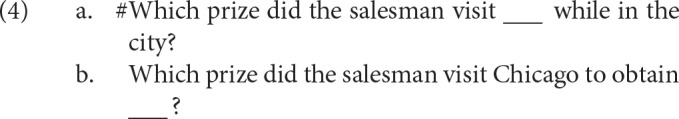


There is a wealth of cross-methodological evidence that comprehenders actively construct filler-gap dependencies: electrophysiology using EEG (Garnsey et al., 1989), eye-tracking while reading paradigm (Traxler and Pickering, 1996), eye-movements in the visual world paradigm (Sussman and Sedivy, 2003), self-paced reading (Lee, 2004), among many others. There is also evidence that active dependency formation is cross-linguistically robust: Bangla (Chacón et al., 2016), Chamorro (Wagers et al., 2015), Dutch (Frazier and Flores d'Arcais, 1989), German (Schlesewsky et al., 2000), Italian (Vincenzi, 1991), Japanese (Aoshima et al., 2004), and Russian (Sekerina, 2003), to name a few. There is also evidence that it is robust in both neurotypical and aphasic populations (Dickey et al., 2007).

Actively associating a filler to a gap may be risky because the initial association may be incorrect and thus, needs revision. How can comprehenders minimize these faulty associations? Framed differently, what types of linguistic information do comprehenders use to guide their incremental structure-building? It is perhaps unsurprising that comprehenders can use different classes of linguistic information. They can leverage syntactic information, like word order (Stowe, 1986) or more abstract structural constraints like islandhood (Traxler and Pickering, 1996; Wagers and Phillips, 2009). They can use lexical information, like a verb's argument structure and thematic relations (Trueswell et al., 1994). They can also use morphological information, like grammatical case (Kamide et al., 2003), and prosodic information (Nagel et al., 1994).

More directly related to the present study is the investigation by Wagers et al. (2015) of what is called *wh*-agreement in the generative syntax literature and its contribution in processing filler-gap dependencies in Chamorro, an Austronesian language distantly related to Tagalog. When present, *wh*-agreement provides direct morphological evidence for the presence of a filler-gap dependency and for the grammatical function of the gap (i.e., whether it is the subject or the object). Wagers et al. found that this piece of morphological information modulated how actively comprehenders constructed dependencies. Only when the verb exhibited overt *wh*-agreement did the comprehenders actively construct filler-gap dependencies. When the verb could have *wh*-agreement but did not, dependency formation was delayed. In the present study, we ask how Tagalog comprehenders use similar morphological information to construct filler-gap dependencies. Like Chamorro, Tagalog exhibits rich verbal morphology that is intimately correlated with which argument can participate in such dependencies. Unlike Chamorro, this piece of morphology appears in both filler-gap and non-filler-gap contexts.

### 2.2. Voice Cross-References the Nominative Argument and Interacts With Filler-Gap Dependency Formation

The basic word order of Tagalog is verb-initial. The order of the elements after the verb is relatively free (Schachter and Otanes, 1983), though there are two competing pressures, agent-first and nominative-last, that could affect the relative order of elements after the verb (Kroeger, 1993). What is crucial to the present study is that verbs typically carry what is often called voice morphology, which always cross-references the nominative argument of the clause that is marked by *ang* [aŋ]. Throughout, the argument that voice morphology cross-references will be in bold and the agent will be underlined.

Consider the sentences in (5). They have the same event structure—a man, a fish, and a store are involved in a buying event, and the thematic relations assigned by the verb to its arguments remain consistent throughout. Notice that the verb form changes and the case markers of the arguments vary depending on the verb form.^1^ In (5-a), the verb exhibits agent voice (AV); and the agent *lalaki* “man” is marked nominative, and the patient *isda* “fish,” genitive, with *ng* [naŋ]. In (5-b), the verb exhibits patient voice (PV); and the patient is now marked nominative, and the agent, genitive. Note that the term voice should not be conflated with the active-passive alternation in English and other more familiar languages (Ross and Teng, 2005; Foley, 2008).


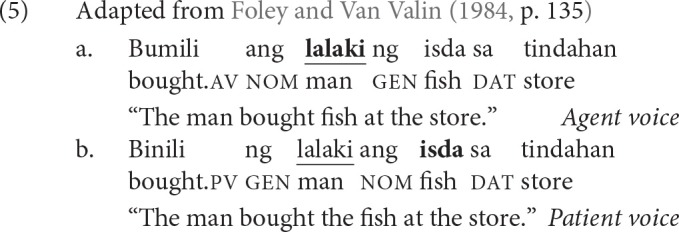


Voice morphology interacts with filler-gap dependency formation because only the noun that is cross-referenced by voice can be displaced (Keenan and Comrie, 1977; Schachter, 1977; Ceña, 1979; Kroeger, 1993; Aldridge, 2002, 2012, 2017; Rackowski and Richards, 2005; Kaufman, 2009; Law, 2016). This interaction is often referred to as the restriction on Ā-extraction in the generative syntax literature. Throughout, we refer to it simply as the displacement restriction to be more descriptive, and restate it in (6) for simplicity.


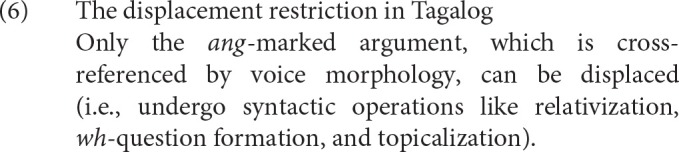


In (7), we show how voice morphology interacts with filler-gap dependency formation. When the verb exhibits AV, only the agent *babae* “woman” can be displaced, as in (7-a). The patient *baro* “dress” cannot be displaced, as in (7-b). In contrast, when the verb exhibits PV, only the patient “dress” can be displaced, as in (7-c). The agent “woman” cannot be displaced, as in (7-d). The star in front of a sentence (“^*^”) indicates ungrammaticality given the intended interpretation.


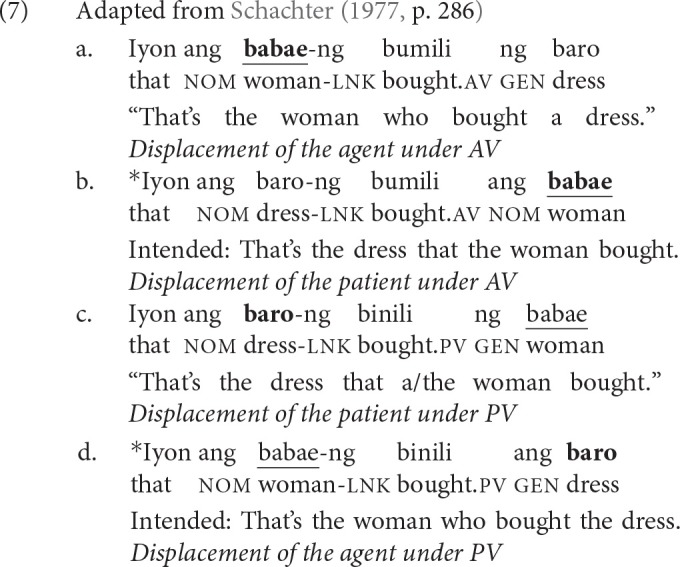


Since post-verbal word order is relatively free, it is difficult to ascertain where the exact location of the gap is. What is crucial for the present study is that voice morphology always precedes the gap. This early availability of voice, along with the other classes of information encoded on the verb, could potentially be leveraged by comprehenders in real-time filler-gap processing. In what follows, we present a generalized schema of how voice could be used by an incremental comprehender and provide a walkthrough of how this schema applies by looking at examples of plausible and implausible displacements in the language.

### 2.3. A Hypothesis: Voice Allows Comprehenders to Commit to an Interpretation Upon Encountering the Verb

Because there is a one-to-one mapping between voice morphology and what is considered to be a licit interpretation of a filler-gap dependency, we hypothesize that voice morphology allows Tagalog comprehenders to commit to an interpretation as early as the verb. Upon encountering the verb, comprehenders already have information about the event structure and by virtue of the voice morphology, the thematic role of the filler. At this point, they should have enough information to evaluate the thematic fit of the filler.

**Figure 1:**
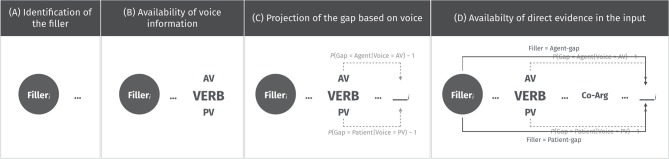
A schema of how a comprehender could use voice to guide her emerging interpretation. In **(A)**, an incremental comprehender recognizes that she is in a filler-gap dependency. In **(B)**, she encounters the verb with the voice morphology. In **(C)**, she projects a gap-site based on the information provided by voice along with the sub-categorization information provided by the verb itself. In **(D)**, she encounters all the bottom-up information in the form of the co-argument that confirms or disconfirms her prediction.

In Figure 1, we provide a generalized schema of how an incremental comprehender could use voice to commit to an interpretation upon encountering the verb in a filler-gap context. In panel A, an incremental comprehender recognizes that a filler-gap dependency is involved. There are various cues that she could use to infer this: (i) word order: she had just encountered a noun phrase before a verb in a verb-initial language; and (ii) overt markers of filler-gap dependencies: immediately after the noun phrase she had just encountered, she may encounter a particle—like another *ang*, for example—that implicates filler-gap dependencies. In this case, the second *ang* signals an upcoming cleft (i.e., *It was X that…*). In panel B, she encounters the verb with the voice morphology. In panel C, she projects a gap-site based on the information provided by voice along with the sub-categorization information provided by the verb itself. Since post-verbal word order is relatively free, it is difficult to ascertain where the exact location of the gap is. Her projection therefore need not be about its exact location but rather about the features that the gap must have to ensure a successful parse. Finally, in panel D, she encounters all the bottom-up information in the form of the co-argument that confirms or disconfirms her expectation.

Next, we illustrate how the schema is applied more concretely by looking at examples of displacements that are plausible and implausible. Approximate time points in a parse are indicated by circled numerals, which correspond to when the comprehender encounters crucial cues. First consider the sentence in (8).


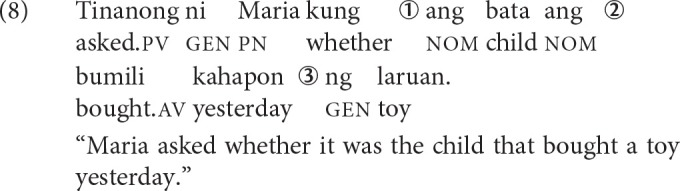


At the complementizer *kung* (before time point ➀), the comprehender knows that she is in an environment that hosts embedded questions. Whether there will be a filler-gap dependency involved remains unclear since *kung* is compatible with both polar (yes-no) and *wh*-questions. At time point ➀, upon encountering the second *ang*, she knows that she is in a cleft construction and thus, there is a gap downstream that must be licensed. Moreover, she has clear evidence about what the filler is, *ang bata* “the child.” At this point, the gap can be linked to the filler, although she has no evidence about its role in the sentence yet. She knows that there are unresolved requirements: (i) to associate the gap to a position with a specific thematic role, and (ii) if applicable, to have a co-argument to satisfy the verb's subcategorization requirement.

At time point ➁, encountering the verb instantiates the event structure and crucially, by virtue of the voice morphology, the thematic role of the filler. She sees that the verb is semantically transitive and thus, needs to have another argument downstream. She also sees that the verb exhibits AV and consequently, projects a gap corresponding to the agent of the verb, and resolves the open requirement from time point ➀ by linking the gap to the agent of *bili* “buy.” Alternatively, she can be conservative and wait until the co-argument is encountered before making any commitments in order to reduce the risk of faulty associations. At time point ➂, upon encountering *laruan* “toy,” she satisfies the subcategorization requirement of the verb. If she was conservative at time point ➁, she now has all the information about how to link the filler and the gap.

Provided in (9) is a sentence that is minimally different from (8). This is a semantically ill-formed sentence, as indicated by the pound sign (“#”) in front. Instead of encountering the filler *ang bata* “the child” at time point ➀, she encounters *ang laruan* ‘the toy.'


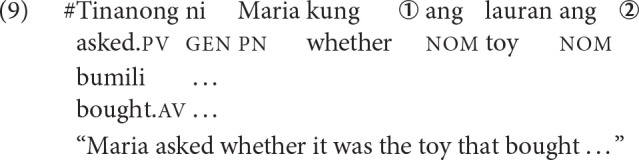


At time point ➁, she sees that the verb is semantically transitive and thus, needs to have another argument downstream. She also sees that the verb exhibits AV and consequently, projects a gap corresponding to the agent of the verb, and resolves the open requirement from time point ➀ by linking the gap to the agent of the verb. Alternatively, she can be conservative and wait. If she does link the filler with the gap, then this should lead to a plausibility mismatch effect. The filler “toy” is a poor fit for a gap corresponding to the agent of “buy”.

The hypothesis presented above crucially hinges on the assumption that voice as a cue has high validity in filler-gap contexts. Cue validity is the conditional probability that an object falls in a particular category, given a particular cue (Beach, 1964; Rosch and Mervis, 1975). Here, what is of interest is the conditional probability of a gap corresponding to a particular form of voice morphology. In other words, we are assuming that there is a one-to-one mapping between voice morphology and what is considered to be a licit interpretation of filler-gap dependencies. We are also assuming that voice is used consistently across different types of dependencies. We summarize our assumptions in (10).


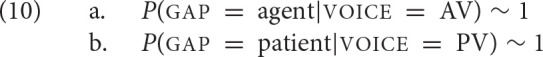


There are reasons to believe that the displacement restriction in (6) might not be as robust as previously documented. First, Ceña and Nolasco (2011, 2012), linguists from the University of the Philippines, have documented that agents may be displaced even when the verb exhibits PV. These works remain largely undiscussed because they were written in Tagalog. Second, we have collected many naturally occurring data from various sources online, ranging from newspapers to Youtube videos, that are consistent with Ceña and Nolasco's descriptions. Finally, our fieldwork has also been consistent with these descriptions. In experiment 1, we are interested in determining whether the assumptions of our hypothesis are robust. Framed differently, we ask whether the received generalization about the displacement restriction in Tagalog is robust.

## 3. Experiment 1: Assessing the Robustness of the Restriction

The main goal of experiment 1 is to assess the robustness of the received generalization about the displacement restriction in Tagalog and see whether the assumptions of the hypothesis we presented in section 2.3 are tenable. In Table 1, we schematize the restriction and frame it in terms of *Match*. Match refers to when the displaced argument is cross-referenced by voice (i.e., displacements of the agent under AV and displacements of the patient under PV). On the other hand, mismatch refers to when the displaced argument is not cross-referenced by voice (i.e., displacements of the patient under AV and displacements of the agent under PV).

**Table 1:** Schematization of the displacement restriction.

	**AV**	**PV**
Match	✓ (7-a)	✓ (7-c)
Mismatch	✗ (7-b)	✗ (7-d)

*Match is when the displaced argument is cross-referenced by voice. Mismatch is when the displaced argument is not. A “✓” reflects acceptable displacements; a “✗” reflects unacceptable displacements*.

To assess the robustness of the restriction, we operationalized voice's cue validity as the participants' ability to detect the illicitness of displacement where the displaced argument is not cross-referenced by the voice (i.e., mismatch), scaled against their ability to detect the licitness of displacement where the displaced argument is cross-referenced by the voice (i.e., match). We manipulated the voice exhibited by the verb and the displaced argument in the three types of filler-gap dependencies: *wh*-questions, relative clauses, and *ay*-inverted sentences (a construction akin to English topicalization). In short, we found that across the three types of dependencies, Tagalog comprehenders were extremely sensitive to mismatches between voice and the displaced argument when the verb exhibited AV. However, their sensitivity varied when the verb exhibited PV. They were the most sensitive in *wh*-questions, and then the least sensitive in *ay*-inverted sentences and relative clauses.

### 3.1. Participants

We recruited 64 speakers from the University of the Philippines—Diliman. They ranged from 18 to 48 years of age (*M* = 26, *SD* = 6.5), and all lived in and around Metro Manila at the time of testing. They received a Starbucks gift certificate valued at 200 PHP for participating in the study.

Our recruitment criteria were fairly lax. Participants needed to (i) be 18–40 years of age; (ii) live in and around Metro Manila at time of testing; and (iii) use Tagalog every day. As one of the reviewers pointed out, people from all over the Philippines live in Metro Manila and most have some proficiency of Tagalog. The laxness did lead to the inclusion of participants who were also exposed to or possibly even speak other Philippine languages (e.g., Cebuano, Ilokano, etc.). While some would argue that this introduced significant variance in the design of our study, this inclusion does not pose any serious concerns. Most Filipinos whose native language is not Tagalog are early sequential bilinguals. They first learn the language of the region or province where they live, and upon entry into the educational system (around age 5), they learn Tagalog and English (Galang, 1988, 2001). That they were in Metro Manila at the time of testing ensured the active use of Tagalog in their daily lives. We view this inclusion positively because it reflected the social reality of who uses Tagalog in the Philippines, thereby increasing our study's ecological validity.

### 3.2. Materials

The experiment employed a 2 × 2 design, crossing whether the verb exhibits AV or PV (Voice: av, pv), and whether the displaced argument is cross-referenced by voice or not (Match: +match, −match). For our experimental items, we created three sets of 32 items involving wh-questions, relative clauses, and ay-inversion (Type: wh, ay, rc). These items were randomized by another set of 32 items that did not involve displacement. Each item was distributed evenly via Latin square, counterbalanced by the four constructions. Apart from the items involving no displacement, no additional fillers were included. Table 2 schematizes the experimental items. Refer to the Supplementary Material (Data Sheet 1) for the experimental items.

**Table 2:** Schematization of the items used in the acceptability judgment experiments.

**TYPE**	**VOICE**	**MATCH**	
WHQ	AV	+	Which Agent	ang	Verb.AV	XP	GEN-Patient	…
	AV	−	Which Patient	ang	Verb.AV	XP	NOM-Agent	…
	PV	+	Which Patient	ang	Verb.PV	XP	GEN-Agent	…
	PV	−	Which Agent	ang	Verb.PV	XP	NOM-Patient	…
RC	AV	+	… Agent	na	Verb.AV	XP	GEN-Patient	…
	AV	−	… Patient	na	Verb.AV	XP	NOM-Agent	…
	PV	+	… Patient	na	Verb.PV	XP	GEN-Agent	…
	PV	−	… Agent	na	Verb.PV	XP	NOM-Patient	…
AY	AV	+	NOM-Agent	ay	Verb.AV	XP	GEN-Patient	…
	AV	−	NOM-Patient	ay	Verb.AV	XP	NOM-Agent	…
	PV	+	NOM-Patient	ay	Verb.PV	XP	GEN-Agent	…
	PV	−	NOM-Agent	ay	Verb.PV	XP	NOM-Patient	…

*Items in WH, RC, and AY manipulated VOICE (AV, PV), and MATCH (+, −)*.

### 3.3. Procedure

The experiment was a pen-and-paper acceptability judgment study. Participants rated the acceptability of sentences using a 7-point Likert-type scale, with 1 being hindi mabuti “not acceptable” (literal: not good) and 7 being mabuti “acceptable” (literal: good).

### 3.4. Data Analysis

We estimated cumulative link mixed models using the R-package Ordinal (Christensen, 2018). We used their ratings as the dependent measure, and the fixed effects were Voice (av, pv), Match (+match, −match), Type (whq, rc, ay), and their interactions. Voice and Match were sum-coded, such that av and +match mapped to the negative coefficients. Type was coded by two coefficients using Helmert contrasts. The first coefficient contrasted whq with ay and rc, where whq is mapped onto the positive coefficient. The second coefficient contrasted rc with ay, where rc is mapped onto the positive coefficient.^2^ We then included the maximal random effects structure justified by the design that allowed the models to converge (Barr et al., 2011; Barr, 2013). The random effects structure included random intercepts for participants and items, and voice, match, and their interaction, and type as random slopes for participants and items.

### 3.5. Results and Discussion

**Figure 2:**
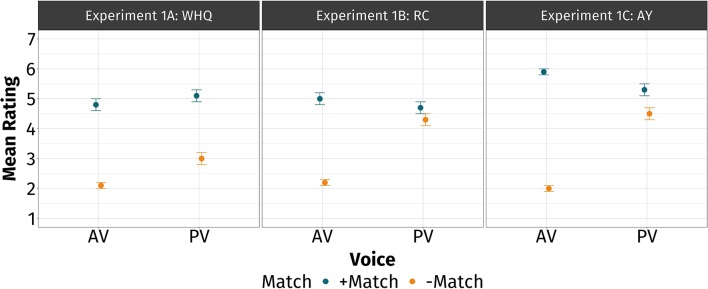
Mean rating by voice, match, and type in experiment 1. In blue are the +match-conditions and in gold are the –match-conditions. Standard errors of the mean are also provided. The leftmost panel represents *wh*-questions (experiment 1A), the middle panel represents relative clauses (experiment 1B), and the rightmost panel represents *ay*-inverted sentences (experiment 1C). The cue validity of voice is estimated as the difference between the blue and gold points. The greater the difference, the higher the cue validity.

In Table 3 and Figure 2, we report the mean ratings by voice, match, and type. The inspection of Figure 2 reveals that AV has high cue validity irrespective of the dependency. The difference between the blue and gold points is consistently large across dependencies. On the other hand, PV had variable cue validity that depended on the dependency. The difference between the blue and gold points varied across constructions. Participants were the most sensitive to mismatches in *wh*-questions, followed by *ay*-inverted sentences, and then by relative clauses. In Table 4, we provide the summary of the estimated model.

**Table 3:** Descriptive statistics of experiment 1.

	**Exp. 1A: WHQ**		**Exp. 1B: RC**		**Exp. 1C: AY**	
	**AV**	**PV**	**AV**	**PV**	**AV**	**PV**
Match	4.83 (0.19)	5.13 (0.18)	4.96 (0.18)	4.72 (0.19)	5.86 (0.14)	5.27 (0.18)
Mismatch	2.09 (0.14)	2.98 (0.18)	2.24 (0.14)	4.34 (0.20)	2.03 (0.15)	4.52 (0.18)

*Mean ratings (with S.E.) by VOICE and MATCH*.

**Table 4:** Summary of cumulative link mixed models in experiment 1.

	**Estimate**	***SE***	**z**
VOICE	1.12	0.20	−5.57^**^
MATCH	−2.88	0.33	8.69^**^
WH vs. RC+AY	−0.48	0.19	2.61^*^
RC vs. AY	−0.58	0.24	2.43^*^
VOICE × MATCH	2.68	0.46	−5.85^**^
VOICE × (WH vs. RC+AY)	0.13	0.28	−0.48
VOICE × (RC vs. AY)	−0.10	0.41	0.25
MATCH × (WH vs. RC+AY)	−1.00	0.28	3.55^**^
MATCH × (RC vs. AY)	1.55	0.41	−3.81^**^
VOICE × MATCH × (WH vs. RC + AY)	−2.17	0.51	4.22^**^
VOICE × MATCH × (RC vs. AY)	0.04	0.79	−0.05

The models included acceptability ratings as the dependent measure, and sum-coded VOICE, MATCH, Helmert-coded Type (WH vs. RC+AY as the first coefficient and RC vs. AY as the second coefficient), and their interactions into the models. ^*^p < 0.05, ^**^p < 0.001.

On average, participants gave higher ratings when the verb exhibited PV (*M* = 4.49) compared to when it exhibited AV (*M* = 3.67). They gave higher ratings when the displaced noun and the verb matched (*M* = 5.13) compared to when they did not (*M* = 3.03). They gave higher ratings to relative clauses and *ay*-inverted sentences combined (*M* = 4.25) compared to *wh*-questions (*M* = 3.76). They gave higher ratings to *ay*-inverted sentences (*M* = 4.43) compared to relative clauses (*M* = 4.06).

These effects were qualified by higher-order interactions. The significant Voice × Match interaction indicated that the difference between −match and +match is greater in av (difference = 3.1) than it is in pv (difference = 1.09) across Type. This suggests that collapsing across the three types of dependencies examined, participants' sensitivity was greater when the verb exhibited AV compared to when it exhibited PV. We take this as evidence that AV has high cue validity across the board.

The significant Match × (wh vs. rc+ay) interaction indicated that the difference between −match and +match is greater in whq (difference = 2.44) than in ay and rc combined (difference = 1.92). This suggests that collapsing across voice, the participants' sensitivity was greater in *wh*-questions than in relative clauses and *ay*-inverted sentences. We take this as evidence for AV and PV having higher cue validity in *wh*-questions than in relative clauses and *ay*-inverted sentences combined.

The significant Match × (rc vs. ay) interaction indicated that the difference between −match and +match is greater in ay (difference = 2.29) than in rc (difference = 1.55). This suggests that collapsing across voice, the participants' sensitivity was greater in *ay*-inverted sentences than in relative clauses. We take this as evidence for AV and PV having higher cue validity in relative clauses than in *ay*-inverted sentences.

Finally, the significant Voice × Match × (wh vs. rc+ay) interaction qualified the two-way interactions found earlier. In other words, the participants' sensitivity in AV was greater than their sensitivity in PV when they encountered relative clauses and *ay*-inverted sentences (difference of difference = 2.72) compared to when they encountered *wh*-questions (difference of difference = 0.59). We take this as evidence that AV has higher cue validity than PV in relative clauses and *ay*-inverted sentences, compared to *wh*-questions where they may be more comparable.

Given what we know about these dependencies, these results are both surprising and unsurprising. It is surprising syntactically and semantically that relative clauses patterned more like *ay*-inverted sentences for two reasons. First, it is widely known in the Austronesian literature that argument *wh*-questions and relative clauses are structurally similar (Potsdam, 2009). In Tagalog, argument *wh*-questions are pseudo-clefts where the *wh*-phrase functions as a predicate nominal and the rest of the material is inside a headless relative clause (Kroeger, 1993; Aldridge, 2002, 2013). For example, the Tagalog equivalent of the question *Which wine did the woman drink?* would literally be *The one that the woman drink is which wine?* In other words, Tagalog argument *wh*-questions are essentially relative clauses. Second, the dependency in relative clauses are thematically more complex than the dependency in *ay*-inverted sentences. The filler in relative clauses serves two thematic roles: one in the embedded clause and another one in the main clause. Meanwhile, the filler in *ay*-inverted sentences only serves one thematic role.

It is also equally unsurprising that relative clauses patterned more like *ay*-inverted sentences. The dependency between the filler and the gap is a direct chain in relative clauses (Law, 2016) and *ay*-inverted sentences (Sabbagh, 2014). On the other hand, owing to the pseudo-cleft structure of argument *wh*-questions, the dependency between the filler predicate and the gap inside the headless relative clause is an indirect one and is mediated by a predication relation (Kroeger, 1993).

More central to the purposes of the present study is that these findings are consistent with the descriptions of Ceña and Nolasco (2011, 2012). The restriction on displacement is not as clean-cut as previously described, which may have consequences for how voice could be used as cue in real-time. Because AV-morphology was a strong diagnostic of a gap corresponding to the agent role, we might expect AV to be a robust trigger for dependency formation in all environments, as previously hypothesized. In contrast, because PV-morphology was less diagnostic of a gap corresponding to the patient role, we might expect PV to trigger less predictive parsing as it becomes less of a diagnostic of a gap corresponding to the patient role. More specifically, as a function of cue validity, we might expect that comprehenders would be the most predictive in *wh*-questions, then least predictive in *ay*-inverted sentences and relative clauses. We summarize these potential consequences in (11).


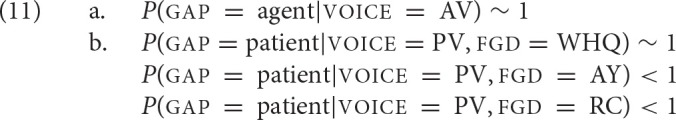


Before we can ask how comprehenders will leverage voice, we first need to establish whether they attend to this cue and actually use it. Cue availability and reliability do not necessarily entail use. We know from the agreement attraction literature that comprehenders are sometimes susceptible to attending to irrelevant cues even when the cue that instantiates the dependency of interest is available (Wagers et al., 2009). We also know from the acquisition literature that comprehenders can ignore very reliable cues and at times, even do so in favor of less reliable ones (Gagliardi and Lidz, 2014). In experiment 2, we address the whether-question by comparing the time course of dependency formation when the verb exhibited voice and when it did not. In experiment 3, we address the how-question by directly comparing the time course of dependency formation when the verb exhibited AV and PV.

## 4. Experiment 2: Isolating the Independent Contribution of Voice

Experiment 2 was designed to isolate the independent contribution of voice when processing filler-gap dependencies. In order to do so, we compared the time courses of dependency formation when the verb exhibited voice and when it did not. Tagalog allows for this comparison to be made because there are grammatical aspects where verbs do not have voice. Crucially, these voiceless paradigms impose comparable restrictions on displacement as when verbs have voice. These voiceless paradigms are discussed in section 4.1.

We used a phrase-by-phrase non-cumulative moving window SMS paradigm (Boland et al., 1990, 1995) in order to detect plausibility mismatch effects that arise when comprehenders link fillers and gaps in *d*-linked *wh*-questions, which are *wh*-questions that have the shape *which N* in English. In this paradigm, sentences were presented one phrase at a time. The phrases did not accumulate across the screen. The participant controlled the presentation rate by pressing the F-key after reading each phrase. The keypress caused the next phrase to appear, unless she pressed the J-key to indicate that the sentence stopped making sense. The participant sets her own criterion for when she will reject the sentence, and following Boland et al. (1995), our linking hypothesis is that plausibility mismatches increase the likelihood that she will press the J-key at a given phrase. Implausible sentences will be non-sensical in a way that only becomes apparent when the participants are able to integrate all of the linguistic material into a coherent whole. When they have indicated that a sentence stopped making sense, it can be inferred that they have assembled the pieces of that sentence into a meaningful unit at that particular point in time.

Like standard self-paced reading paradigm (SPR; Just et al., 1982), SMS forces the comprehender into a strictly incremental mode of processing. However, SMS has some advantages over SPR: (i) SMS data has less variability across conditions compared to SPR data; (ii) it is highly sensitive to anomalies involving the verb's argument structure; and (iii) plausibility effects emerge more locally, typically at the region where incongruity is introduced (Boland et al., 1995; Mauner et al., 1995; Mauner and Koenig, 2000). In the present study, we take advantage of this more fine-grained estimate of where (i.e., at which region) comprehenders commit to an intended parse.

### 4.1. Verbs Without Voice Can Impose Similar Restrictions on Displacement

Even though verbs in Tagalog typically exhibit voice, there are aspectual paradigms in the language where they do not. Crucially, even though verbs in these aspects lack voice, the grammar of the language imposes comparable restrictions on displacement as those that do exhibit voice. Thus, comparing their time courses of dependency formation allows us to approximate the independent contribution of voice in filler-gap processing.

The iterative aspect (glossed as iter) is used to denote repeated or prolonged actions. Voice is optional in iteratives (Schachter and Otanes, 1983, p. 398–399). What is crucial to the present study is that only displacement of the agent is licit when the verb does not exhibit voice, much like when the verb exhibits AV. Because AV and voiceless iteratives impose comparable restrictions on displacement, we argue that this pairwise comparison allows us to isolate the effect of voice in processing filler-gap dependencies. Their comparability is exemplified in (12).


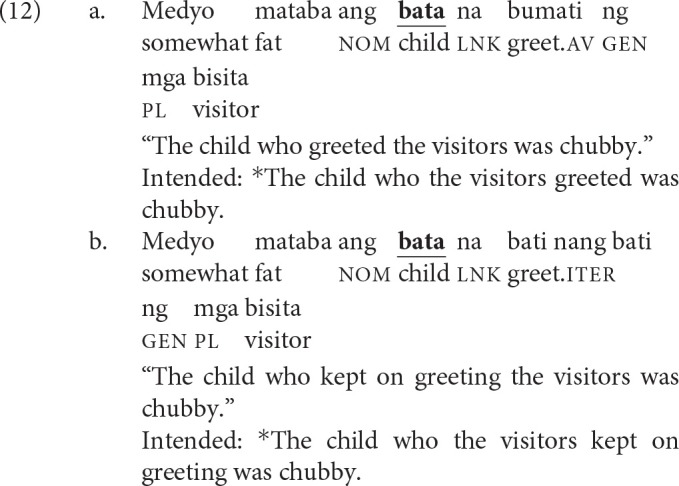


The recent perfective aspect (glossed as rp) is used to denote events that have occurred in the recent past.^3^ This aspect exhibits two quirky properties: (i) the verb exhibits no voice; and (ii) both arguments typically receive the same case marking (Kroeger, 1993; Frazier and Yoshida, 2013). Schematically, a clause in the recent perfective aspect typically has the following shape: Verb—*ng* NP1—*ng* NP2. While these two properties conspire to make a sentence globally ambiguous, there is a robust preference for interpreting NP1 as the agent. Sauppe (2016) provided experimental evidence for this preference. Crucial to the present study is the grammaticization of this preference when what immediately follows is a proper name or a pronoun, as in (13-b). The post-verbal argument can only be interpreted as the agent. Framed differently, in filler-gap dependencies, the filler can now only be interpreted as the patient.


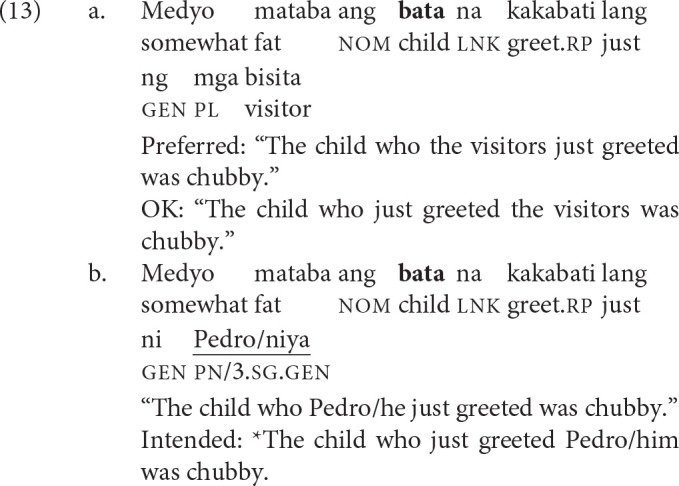


We leveraged (the grammaticization of) this preference to isolate the contribution of PV in processing filler-gap dependencies. As exemplified in (14), PV and recent perfectives now impose comparable restrictions on extraction, with the proviso that the co-argument in the recent perfective be a proper name or a pronoun.


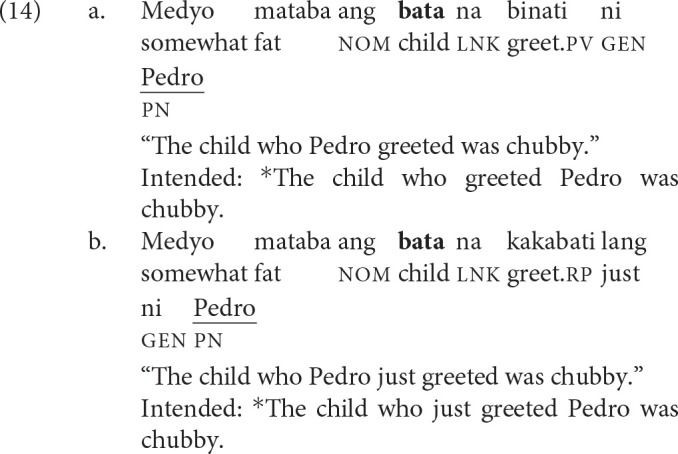


### 4.2. Participants

We recruited 80 speakers from UP Diliman, using the same recruitment criteria discussed in experiment 1. They ranged from 18 to 35 years of age (*M* = 23, *SD* = 4.5), and all lived in and around Metro Manila at the time of testing. They received a Starbucks gift certificate valued at 400 Philippine Pesos (PHP) for participating.

### 4.3. Materials

The experiment employed a 2 × 2 design, crossing whether the verb exhibited voice or not (voice: +voice, −voice) and whether the filler was plausible or not (plausibility: +plaus, −plaus). We created two sets of 12 items involving non-reversible predicates—one for comparing the time course when the verb imposes an agent-only restriction on displacement (experiment 2A), and one for comparing the time course when the verb imposes a patient-only restriction on displacement (experiment 2B). The same 12 verbs were used throughout the experiment but they appeared with different co-argument pairs. Each item was distributed evenly across four lists via Latin Square design. The experimental items were combined together in one session and randomized with 48 filler items that were invariant across all participants. The filler items involved other dependencies, half with voice morphology and half without, and were designed to deter participants from developing a strategy for when to provide a response. Half of the distractors had the plausibility mismatch effect arise before encountering the main verb, and the other half, after encountering the main verb. Provided in Table 5 are sample experimental items in the two sub-experiments. Refer to the Supplementary Material (Data Sheet 2) for the experimental items.

**Table 5:** Sample items for experiment 2.

		**Region**					
		**1**	**2**	**3**	**4**	**5**	**6+**
**Experiment 2A: Comparing AV (+VOICE) and iteratives (–VOICE)**							
+VOICE	+PLAUS	*Aling*	*dalaga*	*ang*	*umiinom parati*	*ng tubig*	…
		which	maiden		drink.AV always	water	
		Which young woman always drinks water …?					
+VOICE	–PLAUS	*Aling*	*tubig*	*ang*	*umiinom parati*	*ng dalaga*	…
		which	water		drink.AV always	maiden	
		#Which water always drinks a young woman …?					
–VOICE	+PLAUS	*Aling*	*dalaga*	*ang*	*inom nang inom*	*ng tubig*	…
		which	maiden		drink.ITER	water	
		Which young woman keeps on drinking water …?					
–VOICE	–PLAUS	*Aling*	*tubig*	*ang*	*inom nang inom*	*ng dalaga*	…
		which	water		drink.ITER	maiden	…
		#Which young woman always drinks water …?					
**Experiment 2B: Comparing PV (+VOICE) and recent perfectives (–VOICE)**							
+VOICE	+PLAUS	*Aling*	*alak*	*ang*	*ininom*	*niya*	…
		which	wine		drink.PV	3sg	
		Which wine did (s)he drink …?					
+VOICE	–PLAUS	*Aling*	*babae*	*ang*	*ininom*	*niya*	…
		which	woman		drink.PV	3sg	
		#Which woman did (s)he drink (earlier) …?					
–VOICE	+PLAUS	*Aling*	*alak*	*ang*	*kakainom lang*	*niya*	…
		which	wine		drink.RP just	3sg	
		Which wine did (s)he just drink …?					
–VOICE	–PLAUS	*Aling*	*babae*	*ang*	*ininom*	*niya*	…
		which	woman		drink.RP just	3sg	
		#Which woman did (s)he just drink …?					

*The first line provides the words in Tagalog; the second line, the glosses; and the third line, the translation*.

### 4.4. Procedure

The experiment was presented using Linger (Rohde, 2003). Participants were first introduced to a moving window SPR to familiarize themselves with the presentation. They were instructed to press the F-key to see the first word of the sentence, and to press the F-key again to see the next word. They were to continue pressing the F-key until they saw the end of the sentence. After some practice SPR-presentations, they were introduced to the stops-making-sense task. They were instructed to read the sentences in the same way as before with one crucial difference: after each phrase, they had the option to abort the presentation by pressing the J-key if the sentence stopped making sense. Aborting the presentation led to the presentation of another sentence. After completing 4 practice items, they started the experiment proper.

### 4.5. Analysis

We couched our analysis in Signal Detection Theory (SDT; Macmillan and Creelman, 2005), a common framework for modeling decision-making processes under uncertainty. For our purposes, the framework can be applied to a comprehender's judgments of whether to reject or continue with an item that may be plausible or implausible. A standard SDT analytical approach to our stops-making-sense task categorizes response behaviors into hits (i.e., correct rejections of implausible items) and false alarms (i.e., incorrect rejections of plausible items). The distributions of the hits and false alarms in a detection task can be used to construct an explicit model of the comprehender's decision process. The comprehender's responses are attributed to a combination of how clearly the comprehender is able to detect signal from noise (i.e., their sensitivity, which is represented by *d'*) and the threshold that they used to make these judgments (i.e., their bias). Thus, one advantage of couching our analysis in SDT is that we can decouple the comprehender's sensitivity from their bias.

To compare the differences in the time course of interpreting sentences with voice morphology and sentences without, we used the sensitivity index in SDT, expressed in *d'*, by scaling the participants' correct rejections of implausible items against their incorrect rejections of plausible items.

At each region, we counted the number of trials where participants had indicated that the sentence stopped making sense. We then added the rejection counts at a particular rejection and the rejection counts at preceding regions. We used these cumulated rejection counts to calculate their cumulative *d'*. We then re-sampled with replacement at the participant-level to create a new sample of participants of the same size (*n* = 80), from which we derived their cumulative *d'* at each region. We repeated this procedure 100,000 times. For each replication, we calculated the mean cumulative *d'* of each condition at each region. We then used these bootstrapped values to estimate the 95% confidence intervals at each region (Macmillan et al., 2004).

At any given region, a *d'* of 0 implies that participants on average did not discriminate implausible sentences from plausible ones. In other words, the rate at which they correctly rejected implausible sentences was comparable to the rate at which they incorrectly rejected plausible ones. A positive *d'* implies that on average, they discriminated implausible sentences from plausible ones, with more correct rejections for the former than misses for the latter. Finally, a negative *d'* implies that on average, they discriminated implausible sentences from plausible ones, but in the opposite direction: the rate at which they incorrectly rejected plausible sentences was greater than the rate at which they correctly rejected implausible ones. In lieu of reporting *p*-values, we provide the 95% CI for each region. If the CI does not contain 0, then the *d'* at that region is significantly different from 0 at the alpha level of 0.05.

To determine whether the presence of voice significantly affected the participants' sensitivity, we subtracted the *d'* of the conditions without voice from the *d'* of the conditions with voice at each region for each replication. We refer to this difference as Δ*d'*. We then calculated the mean Δ*d'* and the 95% CI at each region. A positive Δ*d'* implies that on average, participants had greater sensitivity when the verb exhibited voice than when it did not. A negative Δ*d'* implies the reverse. A Δ*d'* of 0 implies that the presence of voice did not affect their sensitivity. The 95% CIs are interpreted the same way.

### 4.6. Results

**Figure 3:**
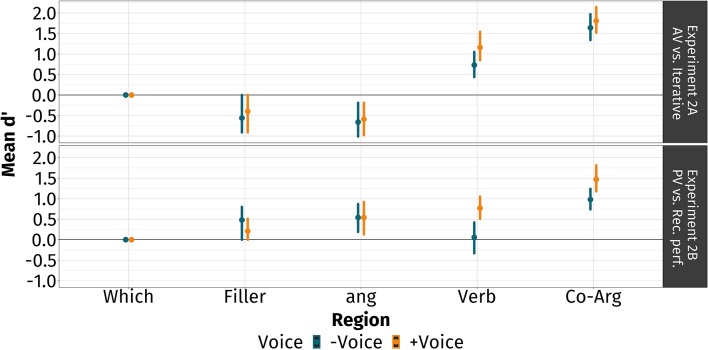
Phrase-by-phrase discrimination scores in experiment 2. Plotted are the mean scores at each region (points) with 95% confidence intervals (bands) derived by a bootstrap over participants. In blue are –voice-conditions and in gold are +voice-conditions. The top panel represents the comparison between AV and iteratives (experiment 2A), and the bottom panel represents the comparison between PV and recent perfectives (experiment 2B). The verb- and co-argument-regions are the critical and disambiguating regions, respectively.

The main findings of experiment 2 are that (i) upon encountering the verb, Tagalog comprehenders actively associated the filler with the gap even before the disambiguating information that came in the form of the co-argument; and (ii) voice enhanced this linkage by allowing them to commit to a correct interpretation upon encountering the verb. In Figure 3, we report the participants' phrase-by-phrase mean cumulative *d'*s and the 95% CI by voice at each region.

#### 4.6.1. Experiment 2A: Comparing AV and Voiceless Iteratives

We did not observe any plausibility effects in the first two regions. At the ang-region, participants were rejecting plausible sentences more than implausible sentences: *d'* = −0.63, 95% CI [−0.98, −0.19]. They showed comparable sensitivity when the verb exhibited voice and when it did not: Δ*d'* = 0.06, 95% CI [−0.23, 0.38].

At the verb-region, they were rejecting implausible sentences more than plausible ones: Δ*d'* = 0.94, 95% CI [0.48, 1.46]. However, they showed even greater sensitivity when the verb exhibited voice compared to when it did not: Δ*d'* = 0.43, 95% CI [0.07, 0.83].

At the co-argument region, they were significantly rejecting implausible sentences more than plausible ones: *d'* = 1.73, 95% CI [1.39, 2.09]. They showed comparable sensitivity when the verb exhibited voice and when it did not: Δ*d'* = 0.17, 95% CI [−0.14, 0.48].

#### 4.6.2. Experiment 2B: Comparing PV and Recent Perfectives

We did not observe any plausibility effects in the first two regions. At the ang-region, they were rejecting implausible sentences more than plausible sentences: Δ*d'* = 0.54, 95% CI [0.18, 0.89]. They showed comparable sensitivity when the verb exhibited voice and when it did not: Δ*d'* = 0.00, 95% CI [−0.26, 0.26].

At the verb-region, they were rejecting implausible sentences more than plausible ones when the verb exhibited voice but not when it did not: *d'* = 0.77, 95% CI [0.51, 1.05] for PV and *d'* = 0.06, 95% CI [−0.33, 0.42] for recent perfectives. This is further corroborated by a significant difference in their *d'*s: Δ*d'* = 0.71, 95% CI [0.30, 1.16].

At the co-argument-region, they were rejecting implausible sentences more than plausible ones: *d'* = 1.23, 95% CI [0.78, 1.75]. However, they showed greater sensitivity when the verb exhibited voice than when it did not: Δ*d'* = 0.49, 95% CI [0.15, 0.86].

### 4.7. Discussion

The goal of experiment 2 was to isolate the independent contribution of voice morphology in processing filler-gap dependencies by comparing the time courses of dependency formation when the verb exhibited voice and when it did not. We found the following. First, upon encountering the verb, participants were actively associating the filler with the gap. This association happened even before they had encountered fully disambiguating information in the form of the co-argument. Second, voice was used as an additional cue to further strengthen the participants' commitment to a correct interpretation.

In experiment 2A, the participants' *d'*s at the verb-region suggest that they were already linking fillers with gaps correctly even in the iterative (–voice). However, they showed greater sensitivity when the verb exhibited AV (+voice). Thus, there was an added benefit to having voice, even when participants had already committed to an interpretation.

In experiment 2B, their *d'*s suggest that only when the verb exhibited PV did they commit to an interpretation. At the co-argument-region, an asymmetric pattern—similar to what was observed with AV and iteratives at the verb-region—emerged: even though their *d'*s suggest they were linking fillers with gaps correctly even in recent perfectives, they showed greater sensitivity when the verb exhibited PV. Thus, there was an added benefit to having voice, even when all of the information to complete the dependency had already been encountered.

There is also the question of why there was a plausibility effect at the ang-region. At this point of the presentation, the verb had yet to be seen, so the rejections could not possibly be attributed to plausibility. We argue that this is in fact an animacy effect. During debriefing and subsequent fieldwork, we learned that some speakers have a restricted environment for the *d*-linked interrogative *alin*. These speakers can only use *alin* with inanimate nouns. In order for an animate noun to be *d*-linked, *sino* “who” needs to be used. For example, these speakers would use *sinong dalaga* “which young woman” instead of *aling dalaga*. In an SMS-task, this would mean that these speakers would judge sentences with *alin* followed by an animate noun as unacceptable and would choose to reject them before encountering the elements downstream. This is entirely consistent with the pattern that we observed. In experiment 2A, plausible sentences (i.e., those with animate fillers) had higher rejection rates than implausible sentences (i.e., those with inanimate fillers). In experiment 2B, implausible sentences (i.e., those with animate fillers) had higher rejection rates than plausible sentences (i.e., those with inanimate fillers). In other words, sentences involving animate fillers had higher rejection rates than those involving inanimate fillers across sub-experiments.

To reiterate, experiment 2 establishes that voice is a cue that comprehenders attend to and use when processing filler-gap dependencies. Even though Tagalog comprehenders actively associated the filler with the gap before encountering the co-argument, having voice on the verb enhanced this association.

## 5. Experiment 3: Comparing the Predictive Value of Voice Cues

It remains an open question as to whether comprehenders use the different types of voice the same way. Experiment 2 did not allow us to directly compare how they used AV and PV because we used different baselines, ones that allowed us to approximate the independent contribution of voice. It also remains an open question as to whether comprehenders use voice the same way across different types of dependencies. Thus, the goal of experiment 3 is to directly compare the time courses of dependency formation when the verb exhibits AV and PV using three different types of filler-gap dependencies.

### 5.1. Participants

We recruited 85 participants from UP–Diliman using the same recruitment criteria as in experiments 1 and 2. They ranged from 18 to 44 years old (*M* = 25, *SD* = 6), and all lived in and around Metro Manila at the time of testing. They received a Starbucks gift certificate valued at 400 PHP for participating in the study.

### 5.2. Materials, Procedure, and Analysis

The experiment employed a 2 × 2 factorial design, crossing whether the verb exhibited AV or PV (voice: av, pv) and whether the filler was globally plausible (plausibility: +plaus, −plaus). We created three sets of 24 items involving non-reversible predicates in *wh*-questions, relative clauses, and *ay*-inverted sentences. The experimental items were combined together in one session and randomized with another set of 24 items involving run-of-the-mill declarative sentences that did not have extraction. Each item was distributed evenly via Latin square design. The same 24 verbs were used throughout the experiment and thus, they appeared four times per participant in different constructions and with different co-argument pairs.

To discourage participants from singling out *wh*-questions as the only non-declarative clause-type, we used embedded questions. To discourage them from creating expectations about when to provide a response, we varied the region where the dependency started: for *wh*-questions, the filler was in region 4; for relative clauses, it was in region 6; and for *ay*-inverted sentences, it was in region 2. Crucially, the distance between the filler, the verb, and the co-argument remained consistent across dependencies. Table 6 provides a sample of our experimental items using *bili* “buy.” Refer to the Supplementary Material (Data Sheet 3) for the experimental items.

**Table 6:** Sample items for experiment 3.

		**Region**					
		**Filler**	**ang**	**Verb**	**XP**	**CoArg**	**…**
**Experiment 3A: AV and PV in** ***wh*****-questions**							
AV	+Plaus	*aling babae*	*ang*	*bumibili*	*gabi-gabi*	*ng alak*	*sa Trinoma*
		which woman		buy.AV	every night	wine	at Trinoma …
		(Ernesto knows) which woman buys wine at Trinoma every night …					
AV	−Plaus	*aling alak*	*ang*	*bumibili*	*gabi-gabi*	*ng babae*	*sa Trinoma*
		which wine		buy.AV	every night	woman	at Trinoma …
		#(Ernesto knows) which wine buys the woman at Trinoma every night …					
PV	+Plaus	*aling alak*	*ang*	*binibili*	*gabi-gabi*	*ng babae*	*sa Trinoma*
		which wine		buy.PV	every night	woman	at Trinoma …
		(Ernesto knows) which wine the woman buys at Trinoma every night …					
PV	–Plaus	*aling babae*	*ang*	*binibili*	*gabi-gabi*	*ng alak*	*sa Trinoma*
		which woman		buy.PV	every night	wine	at Trinoma …
		#(Ernesto knows) which woman the wine buys at Trinoma every night …					
**Experiment 3B: AV and PV in relative clauses**							
AV	+Plaus	*tatay*	*na*	*bumibili*	*taon-taon*	*ng kotse*	*sa Toyota*
		father		buy.AV	every year	car	at Toyota …
		(Rafael thought) the father who buys a car every year at Toyota…(is very small)					
AV	−Plaus	*kotse*	*na*	*bumibili*	*taon-taon*	*ng tatay*	*sa Toyota*
		car		buy.AV	every year	father	at Toyota …
		#(Rafael thought) the car that buys a father every year at Toyota…(is very small)					
PV	+Plaus	*kotse*	*na*	*binibili*	*taon-taon*	*ng tatay*	*sa Toyota*
		car		buy.PV	every year	father	at Toyota …
		(Rafael thought) the car that the father buys every year at Toyota…(is very small).					
PV	−Plaus	*tatay*	*na*	*binibili*	*taon-taon*	*ng kotse*	*sa Toyota*
		father		buy.PV	every year	car	at Toyota …
		#(Rafael thought) the father that the car buys every year at Toyota…(is very small)					
**Experiment 3C: AV and PV in ay-inverted sentences**							
AV	+Plaus	*ang nanay*	*ay*	*bumibili*	*sa KFC*	*ng pagkain*	*kasi*
		mother		buy.AV	at KFC	food	because …
		(Every day,) the mother, she buys food at KFC because (she has no time…)					
AV	−Plaus	*ang pagkain*	*ay*	*bumibili*	*sa KFC*	*ng nanay*	*kasi*
		food		buy.AV	at KFC	mother	because …
		#(Every day,) the food, it buys a mother at KFC because (she has no time…)					
PV	+Plaus	*ang pagkain*	*ay*	*binibili*	*sa KFC*	*ng nanay*	*kasi*
		food		buy.PV	at KFC	mother	because …
		(Every day,) the food, the mother buys it at KFC because (she has no time…)					
PV	−Plaus	*ang nanay*	*ay*	*binibili*	*sa KFC*	*ng pagkain*	*kasi*
		mother		buy.PV	at KFC	food	because …
		#(Every day,) the mother, the food buys her at KFC because (she has no time…)					

*The first line provides the words in Tagalog; the second line, the glosses; and the third line, the translation. Sample items are truncated for reasons of space. Truncated parts are in “()”*.

Experiment 3 used the same procedure as experiment 2. We also used the same dependent measure as in experiment 2 to compare the differences in the time courses of sentences with AV and those with PV when interpreting three types constructions. Their cumulative *d'*s were derived using the same bootstrapping procedure.

### 5.3. Results

**Figure 4:**
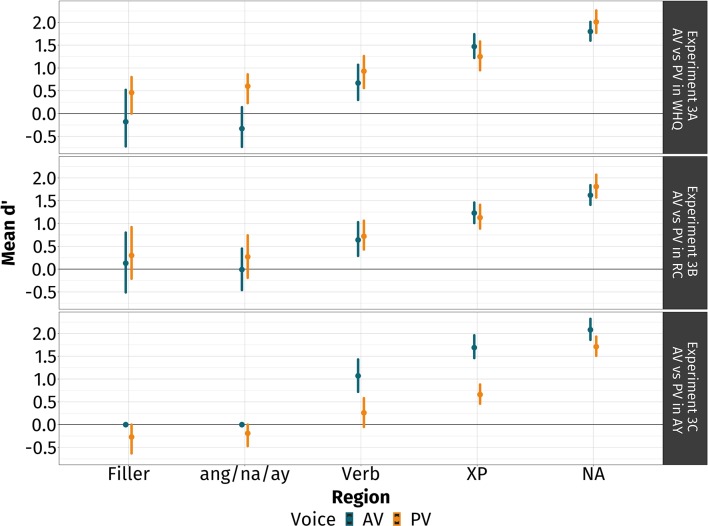
Phrase-by-phrase discrimination scores in experiment 3. Plotted are the mean scores at each region (points) with 95% confidence intervals (bands) derived by a bootstrap over participants. In blue are av-conditions and in gold are pv-conditions. The topmost panel represents the comparison between AV and PV in *wh*-questions (experiment 3A), the middle panel represents the comparison between AV and PV in relative clauses (experiment 3B), and the bottommost panel represents the comparison between AV and PV in *ay*-inverted sentences (experiment 3C). The verb- and XP-regions, and the co-argument-region are the critical and disambiguating regions, respectively.

The main findings of experiment 3 are that (i) Tagalog comprehenders actively associated the filler with the gap even before the fully disambiguating co-argument; and (ii) the way they used voice may be mediated by voice-type and type of filler-gap dependency. In Figure 4, we report the participants' phrase-by-phrase mean cumulative *d'*s and the 95% CI by voice at each region.

#### 5.3.1. Experiment 3A: Comparing AV and PV in *WH*-Questions

We did not observe any plausibility effects at the filler-region. At the ang-region, participants were rejecting implausible sentences more than plausible ones when the verb exhibited PV, but not when it exhibited AV: *d'* = −0.33, 95% CI [−0.73, 0.14] for AV and *d'* = 0.60, 95% CI [0.23, 0.86] for PV.

At the verb-region, they were rejecting implausible sentences more than plausible ones: *d'* = 0.67, 95% CI [0.30, 1.07] for AV and *d'* = 0.93, 95% CI [0.56, 1.26]. Their sensitivity was comparable across different voices: Δ*d'* = −0.26, 95% CI [−0.73, 0.25]. Their rejection behavior at the verb-region persisted into the XP- and co-argument-regions. They were rejecting implausible sentences more than plausible ones, and their sensitivity was comparable across different voices.

#### 5.3.2. Experiment 3B: Comparing AV and PV in Relative Clauses

In experiment 2B, we did not observe any plausibility effects in the filler- and na-regions. At the verb-region, participants were rejecting implausible sentences more than plausible ones: *d'* = 0.64, 95% CI [0.29, 1.03] for AV and *d'* = 0.72, 95% CI [0.43, 1.06] for PV. Their sensitivity was comparable across different voices: Δ*d'* = −0.08, 95% CI [−0.59, 0.42]. Their rejection behavior at the verb-region persisted into the XP- and co-argument-regions. They were rejecting implausible sentences more than plausible ones, and their sensitivity was comparable across different voices.

#### 5.3.3. Experiment 3C: Comparing AV and PV in *AY*-Inverted Sentences

We did not observe any plausibility effects in the filler- and ay-regions. At the verb-region, we found an asymmetry in how participants used voice. They were rejecting implausible sentences more than plausible ones when the verb exhibited AV, but this was not the case when the verb exhibited PV: *d'* = 1.07, 95% CI [0.72, 1.43] for AV and *d'* = 0.26, 95% CI [−0.05, 0.58].

At the XP-region, they were rejecting implausible sentences more than plausible ones across the different types of voice: *d'* = 1.69, 95% CI [1.46, 1.96] for AV and *d'* = 0.66, 95% CI [0.46, 0.88] for PV. The asymmetry found at the verb-region persisted into the XP-region: Δ*d'* = 1.03, 95% CI [0.70, 1.37].

At the co-argument-region, they were rejecting implausible sentences more than plausible ones across the different types of voice: *d'* = 2.08, 95% CI [1.86, 2.32] for AV and *d'* = 1.71, 95% CI [1.51, 1.93] for PV. Their sensitivity was comparable across different voices: Δ*d'* = 0.38, 95% CI [0.13, 0.62].

### 5.4. Discussion

The goal of experiment 3 was to directly compare the time courses of dependency formation when the verb exhibited AV and PV in three different types of filler-gap dependencies. We found the following. First, we replicated the finding from experiment 2 that comprehenders actively associated the filler with the gap even before the fully disambiguating co-argument. Second, we found that the way in which voice was used varied across different voice types and across different dependencies.

The participants' *d'*s at the verb- and XP-regions provide evidence that voice was used a cue when interpreting filler-gap dependencies. Participants were correctly rejecting implausible sentences as early as the verb- and XP-regions, even before they encountered the disambiguating co-argument. These findings replicated experiment 2, and are consistent with studies supporting active dependency formation (Crain and Fodor, 1985; Frazier, 1987; Frazier and Clifton, 1987, *inter alia*). They are also consistent with the idea that comprehenders use various sources of linguistic information—lexico-semantic (Boland et al., 1990, 1995; Trueswell et al., 1994) and morphosyntactic ones (Kamide et al., 2003; Wagers et al., 2015), for example—to form said dependency.

In *wh*-questions and relative clauses, comprehenders used AV and PV symmetrically based on their comparable *d'*s. As early as the verb, voice—both AV and PV—had allowed comprehenders to already commit to the correct interpretation. In contrast, in *ay*-inverted sentences, comprehenders exhibited an asymmetry. Their commitment to the correct interpretation was much earlier when the verb exhibited AV than when it exhibited PV. Their *d'* for AV was significantly positive at the verb-region. Their *d'* for PV, on the other hand, was only significantly positive at the XP-region—and even then, they still showed greater sensitivity when the verb had AV.

In *wh*-questions, comprehenders were also rejecting implausible sentences more than plausible ones when the verb exhibited PV at the ang-region. We argue that this is an animacy effect, much like what we found in experiment 1. The *d*-linked interrogative *alin* is compatible with inanimate fillers, but not animate ones. In an SMS-task, this would mean that these speakers would judge sentences with *alin* followed by an animate noun as unacceptable. This is consistent with the effect that we found for PV at the ang-region.

Why must voice be used differently across different filler-gap dependencies? One straightforward hypothesis is that voice has different cue validities in the dependencies we investigated. The results of experiment 1 indicated that AV-morphology was a strong diagnostic of a gap corresponding to the agent role, while PV-morphology was less diagnostic of a gap corresponding to the patient role. Thus, the results of experiment 3 are consistent with the results of experiment 1.

## 6. General Discussion and Conclusion

We found that Tagalog comprehenders used voice to reliably link the filler to the gap and commit to the correct interpretation before encountering disambiguating information in the form of the co-argument. Thus, we add Tagalog to the general picture that comprehenders actively link the filler to the gap and they can use morphological information, like voice, to do so. However, we saw a wrinkle: the way in which comprehenders leveraged voice as a cue was mediated by the type of filler-gap dependencies.

In what follows, we first discuss the level of granularity of the information about voice that comprehenders use in real-time by relating the results of experiments 1 and 3. We discuss reasons why relative clauses patterned like *wh*-questions, and why they did not pattern like *ay*-inverted sentences. We then look at the asymmetries that we found in experiment 3 and view them as a window to what other classes of information could interact when comprehenders process filler-gap dependencies. When Tagalog comprehenders incrementally interpret these dependencies, they engage with different classes of linguistic information that overlap in time and priority, including (but not limited to) information about the structural similarities or the thematic complexity of the dependencies involved, the relative frequency of the different types of voice in the language, and finer-grained information like construction-specific cue validity.

### 6.1. Do Tagalog Comprehenders Use Cue Validity When Processing Filler-Gap Dependencies?

We know from experiment 2 that comprehenders are able to use morphological information. The level of granularity that comprehenders actually use in real-time, however, remains an open question. Here we discuss how the results of experiment 1 can shed some light on the results of experiment 3.

The hypothesis we presented in the beginning of section 2.3 of how voice can be used in filler-gap processing operates at the grammar-level, aggregating over the intimate correlations between voice and the extracted nominal in the many filler-gap dependencies encountered in the input. In other words, this could be encoded as coarse-grained information about voice. If comprehenders rely on voice at this level, their behavior in an SMS task should be highly symmetrical. In other words, AV and PV should be leveraged similarly across different types of dependencies since they are a strong diagnostic of a gap corresponding to the agent and the patient, respectively.

On the other hand, if they rely on finer-grained information about voice (i.e., their construction-specific validities), then their behavior in an SMS task should mirror the results of experiment 1. AV had high cue validity irrespective of the type of dependency and thus, should be a robust trigger for dependency formation in *all* environments. Meanwhile, PV had variable, construction-dependent cue validity and thus, should trigger less predictive parsing, as it becomes less diagnostic of a gap corresponding to the patient role. More specifically, PV should be the most predictive in *wh*-questions and least predictive in *ay*-inverted sentences and relative clauses.

The participants' symmetrical discrimination behavior in *wh*-questions (experiment 3A) is consistent with comprehenders relying on either coarse-grained or finer-grained information. Under a view where voice operates at the grammar-level, comprehenders used AV and PV symmetrically by hypothesis. Under a view where voice has construction-specific cue validities, comprehenders used AV and PV symmetrically because both voices have high cue validities. Upon encountering the verb, AV and PV were good diagnostics of gaps corresponding to the agent and the patient, respectively.

When we consider their behavior with regard to the other two dependencies, the predictions of the different levels of granularity of voice are pulled in opposite directions, as seen in Table 7. First, comprehenders used AV and PV symmetrically in relative clauses (experiment 3B). That is, they were reliably committing to the correct interpretation as early as the verb-region. This symmetrical behavior is consistent with the view where voice, by hypothesis, operates at the grammar level. However, this is inconsistent with the view that voice has construction-specific cue validities because comprehenders, when the verb has PV, would be the least predictive in relative clauses, as operationalized in experiment 1. Second, comprehenders used AV and PV asymmetrically in *ay*-inverted sentences. That is, AV allowed them to reliably commit to the correct interpretation as early as the verb-region, while PV only allowed them to reliably commit to the correct interpretation as early as the XP-region. This asymmetrical behavior is inconsistent with the view where voice operates at the grammar level. However, this is consistent with the view that voice has construction-specific cue validities. In experiment 1, we found that AV has higher cue validity than PV in this dependency. It is unsurprising then that participants exhibited earlier sensitivity to implausibility when the verb had AV compared to when it had PV.

**Table 7:** A summary of their discrimination behavior in experiment 3 by the different levels of granularity of voice morphology.

	**WHQ**	**RC**	**AY**
General (coarse-grained)	✓	✓	✗
FGD-specific (fine-grained)	✓	✗	✓

*A “✓” represents consistency of the results with the predictions for that level of granularity. A “✗” represents inconsistency of the results with the predictions for that level of granularity*.

We argue that comprehenders are sensitive to the construction-specific cue validities of voice. Under this view, their behavior in relative clauses becomes curious. We would expect comprehenders to use voice asymmetrically in a way that closely resembled their discrimination behavior in *ay*-inverted sentences at the very least. However, their discrimination behavior in experiment 3B suggested that they used voice symmetrically in a way that more closely resembled their behavior in *wh*-questions. Even though comprehenders ultimately used AV and PV symmetrically, there is evidence for an asymmetry, comparable to the one seen in *ay*-inverted sentences, in what we refer to as the participants' unrejected reading times. These are the reading times of the trials where the participant chose to continue reading. Refer to the Supplementary Material (Data Sheet 4) for the analyses of their unrejected reading times.

In experiment 3B, comprehenders exhibited a greater plausibility mismatch effect when the verb exhibited AV at the XP- and co-argument-regions. Their unrejected reading times were longer in the implausible conditions compared to their reading times in the plausible conditions, especially when the verb exhibited AV. We argue that participants were sensitive to the plausibility mismatch even if they had decided to delay the rejection of an implausible item at these regions. This finding supports the claim that comprehenders are sensitive to FGD-specific cue validities of voice, but their ability to integrate fine-grained information in real-time could be modulated by other classes of information, like structural similarity or thematic complexity, for example.

### 6.2. (A)symmetries as a Window to What Classes of Information Interact

Comprehenders can prioritize different types of cues (Tamaoka et al., 2005) and they can even ignore very reliable cues in favor of less reliable ones (MacWhinney et al., 1984; Gagliardi and Lidz, 2014). Here, we look at how fine-grained information like cue validity interacts with other classes of linguistic information available to the comprehender. Below we discuss the apparent (a)symmetries we found in experiment 3 and use them as windows to these interactions.

There are (at least) two ways of accounting for the curious case of the participants' behavior in relative clauses. The first way is to offer an explanation as to why relative clauses patterned like *wh*-questions in experiment 3—despite having very different cue validities for PV, as operationalized in experiment 1. Structural similarity could be a reason why relative clauses patterned like *wh*-questions in experiment 3 despite the results of experiment 1.

One construal of structural similarity is the relatedness of the constructions involved. As mentioned earlier, argument *wh*-questions and relative clauses are structurally similar in Tagalog, since argument *wh*-questions are essentially relative clauses (Kroeger, 1993; Aldridge, 2002, 2013). On the other hand, *ay*-inverted sentences are not related to the other two. Another construal of structural similarity is the similarity in clausal complexity, defined here as the number of clauses involved in the dependency. Both *wh*-questions and relative clauses involve bi-clausal structures and thus have comparable clausal complexities. On the other hand, *ay*-inverted sentences only involve mono-clausal structures (Kroeger, 1993; Sabbagh, 2014). Under either construals of structural similarity, the structural similarity of *wh*-questions and relative clauses was prioritized over construction-specific cue validities, and thus exerted a more powerful influence on the participants' response behavior.

The second way of accounting for the curious case of relative clauses is to offer an explanation as to why relative clauses did not pattern like *ay*-inverted sentences in experiment 3—despite having very comparable cue validities in experiment 1. Thematic complexity could be a reason why relative clauses did not pattern like *ay*-inverted sentences despite the results of experiment 1. As mentioned before, the filler in relative clauses serves one thematic role in the matrix clause and another thematic role in the embedded clause. In contrast, the filler in *ay*-inverted sentences serves only one thematic role. Under this view, the thematic requirements of the filler was prioritized over construction-specific cue validities, and thus exerted a more powerful influence on the participants' response behavior.

In *wh*-questions, even though comprehenders ultimately used AV and PV symmetrically, there is evidence for an asymmetry in their unrejected reading times. At the co-argument-region, their unrejected reading times were longer in the implausible conditions compared to their reading times in the plausible conditions, especially when the verb exhibited PV. In other words, upon encountering the disambiguating co-argument, comprehenders were more sensitive to plausibility mismatches when the verb exhibited PV. This difference cannot be attributed to construction-specific cue validities since AV and PV are equally valid in *wh*-questions. Thus, something else must be underlying this effect. One potential explanation for this is the relative frequency of AV and PV in the language. Corpus studies have found that PV is more frequent than AV in transitive sentences (Cooreman et al., 1984; Garcia et al., 2018). One way to reframe this difference in relative frequency is that comprehenders have more experience integrating incoming information when the verb has PV. Crucially, the plausibility mismatch effect that we found at the co-argument restriction is consistent with the view that construction-specific cue validities were prioritized over information about the relative frequencies of voice.

### 6.3. Issues and Future Directions

Ideally, we would have conducted corpus-based analyses to derive construction-specific cue validities, in the same vein as the first study of Garcia et al. (2019b) but with filler-gap dependencies. However, for practicality reasons, we used the results of our judgment study in experiment 1 to estimate voice's cue validities. In order to use a corpus to calculate the validity of voice as a cue to detect when the extracted nominal is not cross-referenced by the voice on the verb, the corpus would need to be part-of-speech tagged, as well as be dependency parsed. We do not know of any corpora that satisfy these requirements.

We related these judgment-estimated values to their behavior in an SMS task. Given the obvious differences between the two experiments, it is perhaps unsurprising if these two tasks elicited different ways voice is to be leveraged as a cue to guide interpretation. Perhaps their behavior in the SMS task was a reflection of how voice could be used when comprehenders were forced into a strictly incremental mode of parsing with no option to backtrack. On the other hand, their behavior in an untimed judgment study was a reflection of how voice could be used to make terminal state judgments, that is, when all of the bottom-up information became available to the comprehender. We are well aware that our judgment study was an imperfect proxy for estimating real-time construction-specific cue validities. We have no way of completely ruling out this possibility.

Given the nature of our experimental items, it is difficult to adjudicate between the two construals of structural similarity discussed in section 6.2. In future studies, we could control for the number of clauses involved in the dependencies and isolate the effect of the relatedness of the constructions on the participants' behavior by investigating the participants' behavior in long-distance *ay*-inversion. In (15-a), we provide the basic word order of a sentence involving the verb *kailangan* “need.” It takes a complement clause, demarcated with “[ ],” and inside that complement clause, the nominative argument is in its canonical position. That is, there is no displacement involved. In (15-b), we provide an instance where *sina Ben* undergoes local *ay*-inversion. Our experimental items are more akin to this example. Finally, in (15-c), we provide an instance where *sina Ben* undergoes long-distance *ay*-inversion.


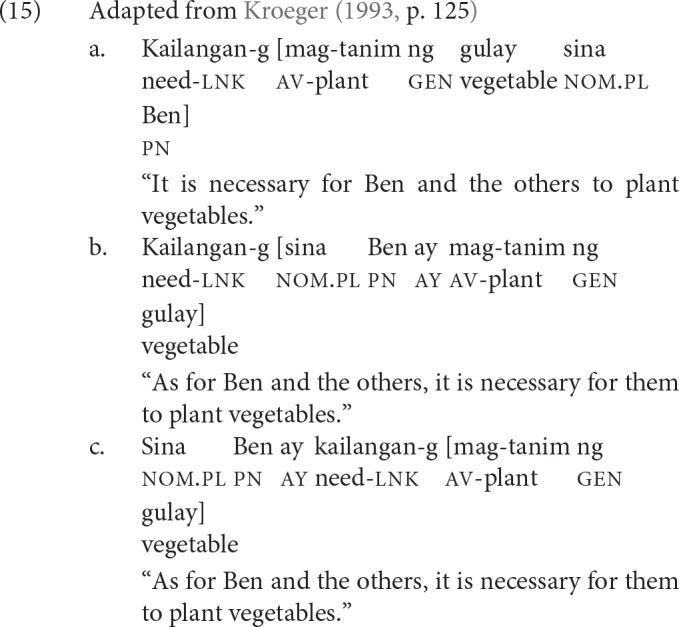


The configuration of *ay*-inversion in (15-c) now matches the clausal complexity of *wh*-questions and relative clauses in experiment 3. This allows us to partial out the contribution of clausal complexity to the participants' response behavior. If clausal complexity was the main driving force of why relative clauses and *wh*-questions patterned together in experiment 3, then we expect to see long-distance *ay*-inversion to pattern like the other two now that the number of clauses is held constant across FGDs. On the other hand, if the relatedness of the constructions was the driving force of why relative clauses and *wh*-questions, then we expect to see the same general pattern. Relative clauses and *wh*-questions would still pattern together, to the exclusion of long distance *ay*-inversion.

Similarly, we can also use long-distance *ay*-inversion to see how tenable of an explanation thematic complexity is for explaining why relative clauses did not pattern like *ay*-inverted sentences. Given the nature of our experimental items, the contributions of clausal complexity and thematic complexity on why relative clauses did not pattern like *ay*-inverted sentences were confounded. Were they different because relative clauses involved bi-clausal structures, while *ay*-inverted sentences involved mono-clausal structures? Or were they different because relative clauses assign two roles to the filler, while *ay*-inverted sentences only assign one role? The configuration of *ay*-inverted sentences in (15-c) now matches the clausal complexity of relative clauses, but crucially, no new thematic relations are introduced.

Lastly, as one of the reviewers pointed out, the higher cue validity of AV is compatible with what has been called the “subject preference” in the literature (Bickel et al., 2015). There is a preference to analyze what is initially an ambiguous NP as being a subject, a cover term to refer to the sole argument of an intransitive verb or the agent of a transitive verb. In a sense, the current findings also point in a similar direction. We found that agents in Tagalog are somehow privileged in filler-gap dependency processing, as exemplified by the AV-PV asymmetry in *ay*-inverted sentences—and potentially, relative clauses, as well. A similar asymmetry has been observed by Tanaka et al. (2019) in Tagalog-speaking children's acquisition of relative clauses, and by Bondoc et al. (2018) in the development of the relativization grammar of aphasics. Sauppe (2016) also observed that agents are privileged when processing canonical declaratives in the language. The behavior of intransitive subjects remain unknown because the studies so far—the present study included—have focused only on predicates with two arguments. Note that the higher cue validity of AV is equally as compatible with what has been called the “agent preference” in the literature (Cohn and Paczynski, 2013). Because agent preference is a proper subset of the subject preference, it is difficult to ascertain whether the high cue validity of AV can be attributed to subjecthood or agenthood. In future studies, we could investigate the comprehenders' behavior when processing two different types of intransitives (Perlmutter, 1978): unergatives, whose sole argument is an agent (e.g., *walk*), and unaccusatives, whose sole argument is a patient (e.g., *sleep*). In doing so, we can investigate which preference could be operative in Tagalog and could be a potential explanation for the high cue validity of AV. If the advantage is potentially due to subjecthood, then we would expect unergatives and unaccusatives to pattern together. On the other hand, if the advantage is potentially due to agenthood, then we would expect unergatives to exhibit an advantage over unaccusatives in processing.

## Data Availability Statement

The raw data supporting the conclusions of this article will be made available by the corresponding author, without undue reservation, to any qualified researcher.

## Ethics Statement

The studies involving human participants were reviewed and approved by UCSC Institutional Review Board. The patients/participants provided their written informed consent to participate in this study.

## Author Contributions

JP-G conducted the linguistic fieldwork, administered the experiments, performed the statistical analyses, and wrote the first draft of the manuscript. JP-G and MW contributed to the conception and design of the study, revised the manuscript, and read and approved the submitted version.

### Conflict of Interest

The authors declare that the research was conducted in the absence of any commercial or financial relationships that could be construed as a potential conflict of interest.

## Footnotes

^1^As one of the reviewers pointed out, the case markers *ang* and *ng*, which we have labeled as “nominative” and “genitive,” respectively, do behave differently from nominatives and genitives in more familiar Indo-European languages. Alternatively, *ang* and *ng* can be referred to as the “pivot” and “non-pivot,” respectively, to really highlight said differences (Sauppe, 2016; Tanaka et al., 2019). Nothing crucial hinges on our choice of labels. We remain agnostic about the debate in the generative syntax and syntactic typology literature concerning the morphological alignment of Philippine-type languages.

^2^The first coefficient of type contrasted wh-questions from relative clauses and ay-inverted sentences: is the relationship between the filler and the gap direct or mediated? Unlike wh-questions in English, argument wh-questions in Tagalog use a (pseudo-)clefting strategy. The interrogative phrase serves as the predicate and the remaining material is a headless relative clause (Kroeger, 1993; Aldridge, 2002). Thus, the relation between the filler and the gap is not direct; it is mediated via predication between what is inside the headless relative clause and the pronounced wh-predicate. In relative clauses and ay-inverted sentences, the filler-gap relation is more direct. The second coefficient of type contrasted relative clauses and ay-inverted sentences: does the filler have multiple thematic roles in the clause? In relative clauses, the filler serves one thematic role in the embedded clause and another thematic role in the main clause. In ay-inverted sentences, the filler serves only one thematic role.

^3^The prefix *kaka-* is added to the base form of the verb in the informal register. The prefix *ka-* is followed by the reduplication of the first syllable of the base form of the verb in the formal register. For example, the verb *bati* “greet” is *kakabati* in the informal register and *kababati* in the formal register. The register difference is immaterial to the purposes of the present study. All of our experimental items were in the informal register.
