# Supplementary material for: The Predictive Value of Tagalog Voice Morphology in Filler-Gap Dependency Formation
Source: Front Psychol. 2020 Apr 15;11:517. doi: 10.3389/fpsyg.2020.00517 (PMC7174735; doi:10.3389/fpsyg.2020.00517)
Supplement: Supplementary file 4 [file Data_Sheet_4.pdf]

Supplementary Material

1 EXPERIMENT 3: UNREJECTED READING TIMES

We used two measures to compare the differences in the time course of interpreting sentences with voice morphology and sentences without. The main dependent measure we used was *d'*, which was calculated by scaling the participants' correct rejections of implausible items against their their incorrect rejections of plausible items (Macmillan and Creelman, 2005), and was reported in the manuscript. The secondary measure we used was their reading times of the trials where they chose to continue reading. We refer to this measure as their unrejected reading times throughout.

For any given trial, at each region, participants had the option to either reject or continue reading. On the trials where they chose to continue reading, their unrejected reading time for that particular region was recorded. We used their unrejected RTs as a secondary measure to determine whether participants were sensitive to plausibility mismatches even if they decided to delay the rejection of implausible items. We analyzed their log-transformed RTs at two regions of interest—the Verb- and CoArg-regions—using *lme4* (Bates et al., 2015) in R (R Core Team, 2018). The fixed effects were VOICE (+VOICE, −VOICE), PLAUSIBILITY (+PLAUS, −PLAUS) and their interaction. These factors were sum-coded, such that +VOICE and +PLAUS mapped to the negative coefficients. We included the maximal random effects structure that allowed the models to converge (Barr, 2013; Barr, Gann, and Pierce, 2011). The *p*-values reported were generated using the Satterthwaite's method in the *lmerTest* package (Kuznetsova, Brockhoff, and Christensen, 2017).

One key difference between the cumulative *d'* and unrejected RTs is the degree of commitment to an interpretation that a comprehender must have given what she has encountered so far. When she chose to reject at a region in any given trial, she had committed to a particular interpretation: what continuations were possible and what continuations were not. In contrast, when she chose to continue reading but displayed slower readings times, she might have experienced processing difficulty—due to a plausibility mismatch effect, for example—but she may have been unable or unwilling to fully commit to an interpretation just yet (Mauner and Koenig, 2000). Together, these measures provide a more nuanced picture of the time courses of FGD-processing.

In Table S1, we report the mean unrejected reading times in *ms* by VOICE and PLAUSIBILITY at the critical and disambiguating regions (i.e., the verb-, XP-, and co-argument-regions). In Table S2, we present the estimated models. In the text that follows, we only report significant effects on their unrejected RTs.

1.1 Experiment 3A: Comparing AV and PV in *wh*-questions

At the verb-region, their unrejected RTs indicated that on trials where they chose to continue reading, they took longer when the verb exhibited PV (M = 830 *ms*) than when it exhibited AV (M = 781 *ms*). This effect was qualified by a significant interaction, such that they read implausible sentences significantly slower than plausible ones when it exhibited PV (ΔRT = 89 *ms*) than when it exhibited AV (ΔRT = -56 *ms*).

At the XP-region, their RTs were longer in the implausible conditions (M = 1168 *ms*) compared to the plausible conditions (M = 978 *ms*). At the co-argument-region, their RTs were again longer in the implausible conditions (M = 1343 *ms*) compared to the plausible conditions (M = 969 *ms*). This effect was

qualified by a significant interaction, such that the difference between plausible and implausible sentences was greater when the verb exhibited PV (ΔRT = 447 *ms*) than when it exhibited AV (ΔRT = 299 *ms*). They were significantly faster when reading plausible sentences with PV.

1.2 Experiment 3B: Comparing AV and PV in relative clauses

At the verb-region, their unrejected RTs suggest that on trials where they chose to continue reading, they still detected the plausibility mismatches. Their RTs were longer in the implausible conditions (*M* = 902 *ms*) compared to the plausible ones (*M* = 748 *ms*).

At the XP-region, their RTs were again longer in the implausible conditions (*M* = 1047 *ms*) than in the plausible ones (*M* = 902 *ms*). This effect was qualified by a significant interaction, such that the difference between plausible and implausible sentences was greater when the verb exhibited AV (ΔRT = 222 *ms*) than when it exhibited PV (ΔRT = 78 *ms*).

At the co-argument-region, their RTs were again longer in the implausible conditions (*M* = 1180 *ms*) compared to the plausible ones (*M* = 980 *ms*). This effect was again qualified by a significant interaction, such that the difference between plausible and implausible sentences was greater when the verb exhibited AV (ΔRT = 291 *ms*) than when it exhibited PV (ΔRT = 125 *ms*).

1.3 Experiment 3C: Comparing AV and PV in *ay*-inverted sentences

At the verb-region, their unrejected RTs suggest that on trials where they chose to continue reading, they took longer when the verb exhibited PV (*M* = 831 *ms*) compared to when it exhibited AV (*M* = 754 *ms*).

At the XP-region, their RTs were longer in the implausible conditions (*M* = 1017 *ms*) compared to the plausible ones (*M* = 955 *ms*).

At the co-argument region, their RTs were longer in the implausible conditions (*M* = 1392 *ms*) compared to the plausible ones (*M* = 1168 *ms*).

1.4 Discussion

The goal of experiment 3 was to directly compare the time courses of dependency formation when the verb exhibited AV and PV in three different types of filler-gap dependencies. We found the following. First, we replicated the finding from experiment 2 that comprehenders actively associated the filler with the gap even before the fully disambiguating co-argument. Second, we found that the way in which voice was used varied across different voice types and across different dependencies.

As we saw, the participants' *d*'s at the verb- and XP-regions provide evidence that voice was used a cue when interpreting FGDs. Participants were correctly rejecting implausible sentences as early as the verb- and XP-regions, even before they encountered the disambiguating co-argument. We add that their unrejected RTs provided further evidence that they were using voice as a cue. On trials where participants decided to continue reading, their longer reading times for implausible sentences suggest that they did detect the implausibility of the sentence even before the co-argument was encountered.

When we also consider their unrejected RTs, a more nuanced picture emerges. There was a PV-advantage at the co-argument-region in *wh*-questions and an AV-advantage at the XP-region in relative clauses. We allude to these asymmetries in greater detail in our general discussion.

Table S1. Mean unrejected reading times (*ms*) at the Verb-, XP-, and co-argument-regions by VOICE and PLAUSIBILITY with the standard error of the mean (*SE*) and the difference in reading times (ΔRT: RT_{-VOICE} – RT_{+VOICE}

			Verb-region			XP-region			Co-Arg-region		
	VOICE	PLAUS	*M*	*SE*	ΔRT	*M*	*SE*	ΔRT	*M*	*SE*	ΔRT
Exp. 3A WHQ	AV	+	808	28	-56	1019	37	125	1037	39	299
	AV	–	752	25		1144	47		1336	77	
	PV	+	787	42	89	938	32	248	902	24	447
	PV	–	876	31		1186	44		1349	76	
Exp. 3B RC	AV	+	729	18	64	876	33	222	948	35	291
	AV	–	793	33		1098	47		1239	76	
	PV	+	768	33	239	928	41	78	1009	42	125
	PV	–	1007	177		1006	47		1134	62	
Exp. 3C AY	AV	+	745	33	19	953	37	1	1214	66	240
	AV	–	764	32		954	42		1454	116	
	PV	+	786	28	90	957	55	107	1122	38	225
	PV	–	876	44		1064	41		1347	68	

Table S2. Summary of linear mixed-effects models in experiment 3. The models included the log-transformed unrejected RTs as the dependent measure, and sum-coded VOICE, PLAUSIBILITY and their interaction into the models as fixed effects. Random effects included the maximal structure that converged.

	Verb-region			XP-Region			Co-Arg-Region		
	Estimate	*SE*	*t*	Estimate	*SE*	*t*	Estimate	*SE*	*t*
**Exp. 3A: *Wh*-questions**									
Intercept	6.53	.03	244.26**	6.81	.05	126.98**	6.86	.05	143.69**
VOICE	.04	.02	2.02*	-.006	.02	-.27	-.03	.03	-1.05
PLAUS	.03	.02	1.36	.18	.03	5.83**	.25	.05	5.43**
VOICE×PLAUS	.02	.04	3.86**	.08	.04	1.87†	.11	.05	2.00*
**Exp. 3B: Relative clauses**									
Intercept	6.51	.03	197.94**	6.72	.05	131.20**	6.80	.04	192.58**
VOICE	.02	.02	.80	-.05	.03	-1.57	-.02	.05	-.54
PLAUS	.06	.02	2.64**	.19	.03	5.55**	.19	.03	6.08**
VOICE×PLAUS	.04	.04	.91	-.15	.05	-3.33**	-.12	.06	-2.01*
**Exp. 3C: *Ay*-inverted sentences**									
Intercept	6.50	.03	192.04**	6.72	.05	141.46**	6.92	.06	124.08**
VOICE	.07	.02	3.26*	.02	.03	.74	-.001	.04	-.03
PLAUS	.04	.02	1.95†	.01	.02	4.61**	.17	.05	3.61*
VOICE×PLAUS	.01	.04	.34	.05	.05	1.02	-.004	.06	-.06

†$p < .10$, *$p < .05$, **$p < .001$

REFERENCES

Barr, D. J. (2013). Random effects structure for testing interactions in linear mixed-effects models. *Frontiers in Psychology* 4, 328

Barr, D. J., Gann, T. M., and Pierce, R. S. (2011). Anticipatory baseline effects and information integration in visual world studies. *Acta psychologica* 137, 201–7

Bates, D., Mächler, M., Bolker, B., and Walker, S. (2015). Fitting linear mixed-effects models using lme4. *Journal of Statistical Software* 67, 1–48

Kuznetsova, A., Brockhoff, P. B., and Christensen, R. H. B. (2017). lmerTest package: Tests in linear mixed effects models. *Journal of Statistical Software* 82, 1–26

Macmillan, N. A. and Creelman, C. D. (2005). *Detection Theory: A User's Guide* (Mahwah, New Jersey: Lawrence Erlbaum Associates), 2 edn.

Mauner, G. and Koenig, J.-P. (2000). Linguistic vs. conceptual sources of implicit agents in sentence comprehension. *Journal of Memory and Language* 43, 110–34

R Core Team (2018). *R: A Language and Environment for Statistical Computing*. R Foundation for Statistical Computing, Vienna, Austria
